# Lung Volume Reduction in Advanced Emphysema

**Published:** 2017

**Authors:** Nicholas S Hopkinson

**Affiliations:** NIHR Respiratory Biomedical Research Unit at Royal Brompton and Harefield NHS Foundation Trust and Imperial College London.

Hyperinflation is a cardinal feature of COPD associated with reduced daily physical activity and increased mortality. Although bronchodilators and pulmonary rehabilitation have some effect many patients with COPD remain highly symptomatic.

The most established treatment is lung volume reduction surgery and results from the NETT trial show improved survival in appropriately selected patients with upper lobe predominant disease and low exercise capacity which are sustained out to at least 7 years. There is a Grade A evidence for LVRS in ARTS/ERS COPD guidelines and patients should be being systematically assessed for potential suitability. The risk of adverse outcomes in modern practice appears to be less than in the NETT. The cost/QALY of LVRS is between $40,0000 and $50,000.

Various other techniques are being assessed either to reproduce the effects of surgery with potentially fewer side effects or to enable lung volume reduction in different populations. Endobronchial valves prevent air entering a target lobe causing atelectasis and improving the function of other areas of lung. They do not work in the presence of interlobar collateral ventilation which occurs if the interlobar fissures are not intact. The BeLieVeR-HIFi and Dutch STELVIO studies have shown that valve placement improves lung function, exercise capacity and quality of life. They reduce dynamic hyperinflation, improve oxygen kinetics and chest wall synchrony and may produce a survival benefit. The estimated cost per QALY of endobronchial valves is 25,000 Euros.

Airway coils tension the target area of lung preventing airway collapse and have shown promise in early results although sustained effects are modest. Other approaches are the use of steam to shrink target areas through contraction fibrosis and the use of fibrin glue.

Airway bypass techniques should work in the presence of collateral ventilation but are limited by problems keeping them patent, so benefit is very short-lived.

As the evidence base and experience with patient selection for different therapies develops it is important for patients with potentially treatable emphysema to be discussed in a multidisciplinary meeting including chest physicians, radiologists and surgeons.

